# Umbilical Arteriovenous Malformation in a Healthy Neonate with Umbilical Hernia

**Published:** 2013-07-01

**Authors:** Mariano Marcelo Boglione, Pablo D´Alessandro, Aixa Reusmann, Martín Rubio, José Lipsich, Gustavo Goldsmith

**Affiliations:** Department of Surgery, Hospital Juan P. Garrahan, Argentina; 1 Department of Imaging, Hospital Juan P. Garrahan, Argentina; 2 Department of Neonatology, Hospital Juan P. Garrahan, Argentina

**Keywords:** Arteriovenous fistulae, Umbilical malformation, Neonate

## Abstract

We describe the case of a neonate with an umbilical hernia and persistent wet umbilicus. Examination revealed a pulsatile umbilical cord with palpable thrill. Doppler ultrasound suspected umbilical arteriovenous malformation and contrast-enhanced computed tomography was performed leading to a definitive diagnosis. Surgery was successfully performed on day 27.

## INTRODUCTION

Arteriovenous malformation (AVM) is a congenital abnormal vascular anomaly in which blood flows directly from an artery into a vein in the absence of a capillary network. AVM can usually involve a variety of organs, such as the brain (aneurysm of the vein of Galen malformation), liver (hemangioendothelioma), kidney, pancreas, lung, placenta (chorangioma) and in sacrococcygeal teratoma. Clinical symptoms of AVM are variable, ranging from the asymptomatic to life threatening hemorrhage or congestive heart failure [1-3]. We report herein the case of a neonate with umbilical AVM and umbilical hernia.

## CASE REPORT

A 20-day-old male neonate with an umbilical hernia was referred to our hospital because of persistent wet umbilicus. Umbilical examination showed a 2 cm diameter umbilical hernia and a long epithelialized pulsatile cord with granulomatous tissue at its end (Fig. 1). Palpable thrill was note at the midportion of the umbilical cord. Doppler ultrasonography revealed a mass at the end of the umbilical cord harboring dilated blood vessels with turbulent blood flow (Fig. 2). This vascular mass continued to the umbilical vein, which was dilated and flowed into the liver. Abdominal contrast-enhanced computed tomography revealed umbilical AVM; the umbilical arteries were patent from both internal iliac arteries, flowed into the mass directly at the end of the umbilical cord, and the dilated umbilical vein flowed out from the mass, continuing from the umbilicus to the umbilical part of the portal vein in the liver (Fig. 3, 4). 

**Figure:**
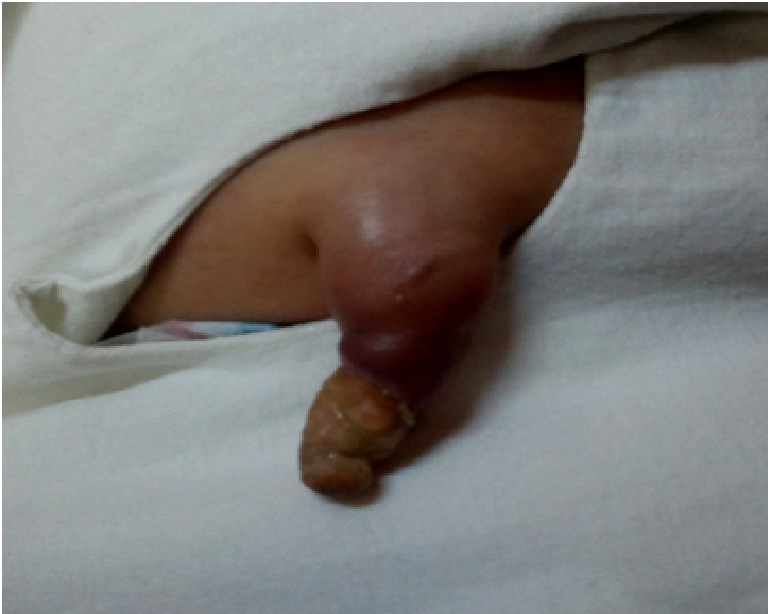
Figure 1: Umbilical hernia and epithelialized pulsatile cord with granulomatous tissue.

**Figure:**
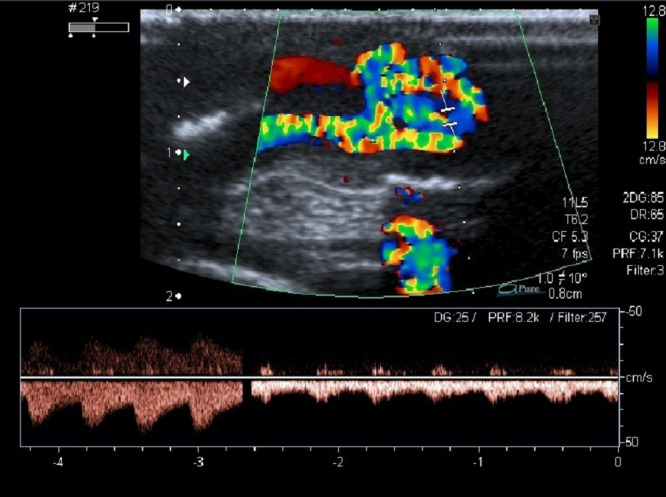
Figure 2: Dilated blood vessels showing turbulent bloodstreams.

**Figure:**
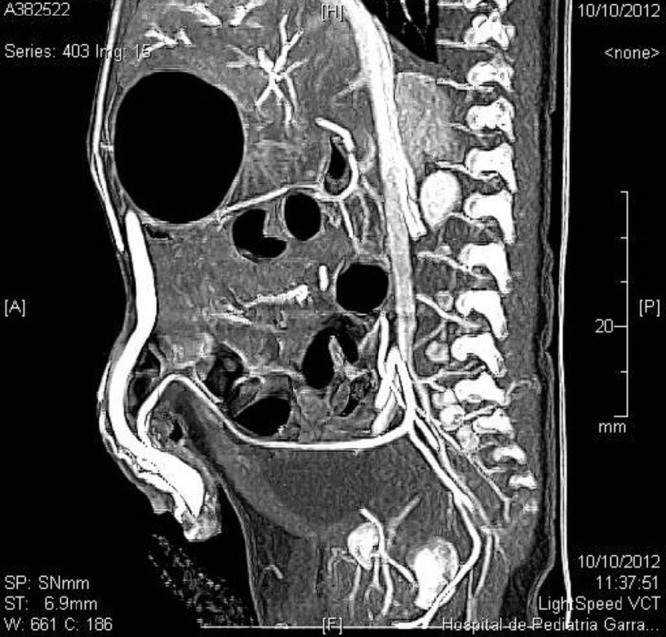
Figure 3: Umbilical arteries arising from both internal iliac arteries, flowing into the mass directly at the end of the umbilical cord.

**Figure:**
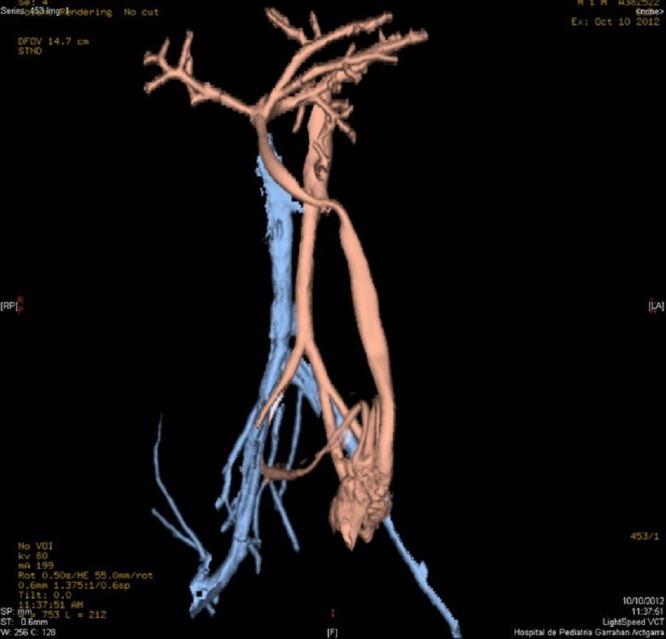
Figure 4: Umbilical AVM with inflow from the umbilical arteries and outflow via the umbilical vein.

Excision of the AVM, closure of the umbilical defect, and umbilicus reconstruction was performed on day 27. Both umbilical arteries and the umbilical vein were ligated at the umbilicus ring level. Post-operative course was uneventful and the patient was discharged three days later. Histological findings were numerous arteries and veins running irregular courses around the two main arteries, with interconnections to blood vessels that it was not possible to differentiate as arteries or veins.

## DISCUSSION

AVM involving the umbilical vessels is rare; both congenital and acquired have been described [4-6]. Reagan et al [4] described arteriovenous fistula between the left umbilical artery and the umbilical vein that most likely occurred as a result of umbilical vein catheterization; this fistula closed spontaneously. An acquired fistula occurring at the site of cord ligation has also been reported [5]. This patient was symptomatic, with congestive heart failure, and required operative resolution. Shibata [1] reported the case of a neonate with umbilical AVM that presented with hemorrhagic shock from massive umbilical hemorrhage; the patient was successfully treated by surgical excision. In our case, AVM was located in the distal portion of the umbilical cord. The patient was asymptomatic, so scheduled surgical excision was performed. Umbilical AVM has been known to cause fetal disseminated intravascular coagulation [7]. The umbilical arteries are formed from portions of the most caudal, ventral segmental arteries, while the umbilical veins are originally paired allantoic vessels. The left umbilical vein eventually fuses with the ductus venosus, and the right vein degenerates. One can speculate that umbilical AVMs result from abnormal anastomoses between unresorbed portions of the ventral segmental arteries and the right umbilical vein. 

Congenital umbilical arteriovenous malformations are exceedingly rare, and its consequences, when present and remain untreated, can be grave. Treatment depends on surgical interruption of the aberrant communication. Most of the reported cases have been successfully treated by complete excision.

## Footnotes

**Source of Support:** Nil

**Conflict of Interest:**None

